# A retrospective study on the application of unilateral epidural anesthesia in older patients with hip fracture

**DOI:** 10.1186/s12877-026-07859-z

**Published:** 2026-06-19

**Authors:** Peng Liu, Xin Shi, Jiang Hu, Jiabing Li, Weimin Liang, Weizhe Jiang, Jingbo Zhao

**Affiliations:** 1 Department of Orthopedic Surgery, Sichuan Provincial People’s Hospital, University of Electronic Science and Technology of China, Chengdu, China; 2 Department of Anesthesiology, Sichuan Provincial People’s Hospital, University of Electronic Science and Technology of China, No. 32 West Second Section, First Ring Road, Chengdu, 610072 China; 3 Department of Anesthesiology, Bazhou District People’s Hospital, Bazhong, Sichuan China

**Keywords:** Unilateral epidural anesthesia, Combined spinal-epidural anesthesia, Hip fracture, Older patients, Hemodynamic stability

## Abstract

**Background:**

We analyzed data from older patients (≥ 65 years) with hip fractures who underwent surgical treatment with unilateral epidural anesthesia (UEA) or combined spinal-epidural anesthesia (CSEA) to compare perioperative outcomes between the two anesthetic techniques.

**Patients and methods:**

One hundred and six older patients with hip fractures who underwent surgery were retrospectively analyzed and stratified into UEA and CSEA groups. The clinical variables investigated included ephedrine use, preanesthetic and postanesthetic hemodynamic heart rate, oxygen saturation changes at 5, 10, 15, and 20 min, complications, duration of hospital stay, mortality, and lower limb function.

**Results:**

The duration of anesthesia in the CSEA group was shorter than in the UEA group (*P* < 0.05). There were no significant differences in sensory block duration or recovery (*P* > 0.05). Systolic blood pressure was significantly higher in the UEA group than in the CSEA group at all post‑anesthesia time points (5, 10, 15, and 20 min) (all *P* < 0.05). There were no significant differences in vasopressor use, hypotension, urinary retention, or phantom limb sensations between the two groups (all adjusted *P* > 0.05). No significant differences were found in sedative use, postoperative complications, duration of hospital stay, critical care admission, mortality, or lower limb function (*P* > 0.05).

**Conclusion:**

Compared with CSEA, UEA was associated with more stable hemodynamics. Despite a longer induction time, UEA represents a viable anesthetic option for older patients with hip fractures. These findings are exploratory and hypothesis-generating due to the retrospective, non-randomized design. Further prospective studies are needed to confirm these results.

## Introduction

Due to population aging, fragility fractures have become a huge burden on healthcare systems and society [1, 2]. Hip fractures, including femoral neck and intertrochanteric fractures, are common in older adults, partly because of fragility associated with osteoporosis and poor response ability in such patients. Older persons are prone to falling while walking, and even a slight external force can lead to hip fractures. They have comparatively weak metabolic activity and poor capacity for tissue repair. A large number of powerful muscle attachment points exist around the hip joint, leading to substantial local stress and poor local blood supply. Once a hip fracture occurs, healing without intervention is difficult.

Hip fracture leads to a dramatic decrease in short- and long-term quality of life, and therefore, has a strong impact on the patient’s activities of daily living and mobility. It has been reported that this is associated with a significant increase in 1-year mortality (11.5%–35.5%) [3–5]. Older patients with hip fractures can benefit from surgery to an extent [6, 7], but this entails inevitable surgical risks, regardless of whether older patients receive general or spinal anesthesia [8, 9]. It is believed that the poor prognosis of hip fractures in older patients results from the combined effects of many factors such as old age, comorbidities, anesthesia, surgery, and complications. Consequently, it is difficult to attribute mortality to a single factor alone. Currently, there are no anesthetic methods or drugs that are particularly suitable for use in older patients [10].

In recent years, unilateral epidural anesthesia (UEA) aims to achieve a predominantly one-sided block rather than a truly selective unilateral block, with relative preservation of contralateral motor and sensory function, and offers advantages such as reduced anesthetic dose, effective sensory blockade, and improved hemodynamic stability [11]. In the current study, based on previous research, anesthetics were injected into the epidural space prior to surgery with the aim of achieving preferential unilateral distribution, as a potential anesthetic approach for older patients with hip fractures.

## Methods and patients

### General information

This retrospective study evaluated 106 older patients (49 male and 57 female) aged 65–94 years with hip fractures who underwent surgery between January 2018 and June 2020 at the Orthopedic Department of our Hospital. Patients were retrospectively classified into two groups according to the anesthesia technique they had received in clinical practice: the unilateral epidural anesthesia (UEA) group (*n* = 42), in which a diluted local anesthetic solution combined with lateral positioning was used to facilitate a predominantly unilateral distribution of the anesthetic, and the combined spinal-epidural anesthesia (CSEA) group (*n* = 64). The choice of anesthesia technique was made by the attending anesthesiologist based on clinical judgment, taking into account patient condition and surgical factors. No strict predefined allocation criteria were applied. Furthermore, the two techniques differed not only in the route of administration but also in anesthetic dose and patient positioning. There were 50 cases of femoral neck fractures, 49 cases of intertrochanteric fractures, and 7 cases of subtrochanteric fractures. The times from fracture to surgery ranged from 1 to 10 days (mean 2.7 days), and body mass index ranged from 14.6 to 28.6 kg/m^2^ (mean 22.8 kg/m^2^). Twelve patients had a history of proximal femoral surgery on the healthy side. Most patients had varying degrees of comorbidities, including hypertension, diabetes, coronary heart disease, arrhythmia (conduction block, atrial premature, ventricular premature, atrial fibrillation), Parkinson’s disease, sequelae of stroke, venous thrombosis, chronic bronchitis, emphysema, and pulmonary heart disease. Among them, 30 patients had one comorbidity, 41 patients had two, and 28 patients had three or more comorbidities (Table 1). The clinical variables investigated included ephedrine usage; preanesthetic and postanesthetic hemodynamic and oxygen saturation (SpO_2_) changes at 5, 10, 15, and 20 min; complications; duration of hospital stay; mortality; and lower limb function. This study was approved by the Ethics Committee of Sichuan Provincial People’s Hospital and conducted under the guidance of the Declaration of Helsinki.

**Table 1:** Characteristics of patients with hip fractures who underwent spinal anesthesia

Characteristic	UEA group (*n* = 42)	CSEA group (*n* = 64)	Chi-square/t	*P*	Adjusted-*P*
Age (years)	72.48 ± 6.55	73.25 ± 6.97	-0.572	0.568	0.850
Gender (female/male)	23/19	34/30	0.027	0.869	0.985
Body mass index (kg/m^2^)	22.62 ± 3.19	22.94 ± 3.80	-0.457	0.649	0.850
Onset time of fracture (days)	2.98 ± 2.80	2.55 ± 2.51	0.823	0.412	0.850
Fracture type (%)			--	0.667	0.850
femoral neck	22/42 (52.38)	28/64 (43.75)			
intertrochanter	17/42 (40.48)	32/64 (50.00)			
subtrochanteric or multiple locations	3/42 (7.14)	4/64 (6.25)			
Surgery type (%)			--	0.187	0.850
prosthetic replacement	24/42 (57.14)	32/64 (50.00)			
fixation with intramedullary nail	13/42 (30.95)	29/64 (45.31)			
fixation with screw	5/42 (11.90)	3/64 (4.69)			
ASA Classification-no./total no. (%)			--	0.700	0.850
I, no systemic disease	0/42 (0)	2/64 (3.13)			
II, mild systemic disease	11/42 (26.19)	21/64 (32.81)			
III, severe systemic disease	26/42 (61.90)	35/64 (54.69)			
IV, severe systemic disease with constant threat to life	5/42 (11.90)	6/64 (9.38)			
Comorbidity burden *n* (%)			--	0.466	0.850
No	1/42 (2.38)	6/64 (9.38)			
1	11/42 (26.19)	19/64 (29.69)			
2	19/42 (45.24)	22/64 (34.38)			
≥ 3	11/42 (26.19)	17/64 (26.56)			
Coexisting comorbidity					
pulmonary disease	27/42 (64.29)	35/64 (54.69)	0.962	0.327	0.850
cardiovascular disease	22/42 (52.38)	30/64 (46.88)	0.308	0.579	0.850
cerebrovascular disease	17/42 (40.48)	29/64 (45.31)	0.241	0.623	0.850
diabetes	8/42 (19.05)	10/64 (15.63)	0.211	0.646	0.850
thyroid disease	1/42 (2.38)	0/64 (0)	--	0.396	0.850
neurological disorders	6/42 (14.29)	5/64 (7.81)	--	0.338	0.850
deep venous thrombosis	3/42 (7.14)	3/64 (4.69)	--	0.679	0.850
abnormal liver function	1/42 (2.38)	3/64 (4.69)	--	1.000	1.000
abnormal kidney function	1/42 (2.38)	1/64 (1.56)	--	1.000	1.000

Data are presented as mean ± standard deviation or *n* (%). Continuous variables were compared using the independent-samples t-test. Categorical variables (fracture type, surgery type, ASA classification, comorbidity burden) were analyzed using Fisher’s exact test for overall constituent proportions; only the *P* value is reported for each category. Each individual comorbidity was tested separately using the chi-square test or Fisher’s exact test, as appropriate. Fisher’s exact test was used when > 1/5 of cells or any cell had a theoretical frequency < 5, with test statistics denoted as “--“. All statistical tests in this table were adjusted for multiple comparisons as a single family using the Benjamini–Hochberg procedure

*UEA* unilateral epidural anesthesia, *CSEA *combined spinal epidural anesthesia, *ASA *American Society of Anesthesiologists Physical Status

### Inclusion and exclusion criteria

Patients were eligible for inclusion if they: (1) had a proximal femoral fracture; (2) were aged ≥ 65 years; (3) sustained the fracture within 2 weeks prior to treatment; (4) had comorbidities such as pulmonary disease, arrhythmia, senile valve disease, or lacunar infarction; and (5) underwent intraspinal anesthesia during surgery. Patients were excluded if they: (1) had a secondary fracture after endoprosthetic reconstruction or intramedullary nailing; (2) had a pathological fracture due to tumor or infection; (3) presented with lower limb neurological dysfunction; (4) had cognitive or psychiatric disorders; (5) had coagulation disorders or underwent emergency procedures (e.g., bone cement implantation syndrome); (6) had incomplete hospitalization data; or patients with failed CSEA or UEA requiring general anesthesia.

### Preoperative preparation

The comorbidities were actively treated. Preoperative requirements included correcting hemoglobin level at > 90 g/L with iron and erythropoietin, maintaining blood glucose at < 8.0 mmol/L 2 h after a meal with hypoglycemic drugs or insulin, maintaining plasma albumin at ≥ 30 g/L via nutritional support or direct intravenous supplementation, maintaining blood pressure at < 160/90 mmHg with β receptor blockers or calcium antagonists, maintaining oxygen partial pressure at ≥ 80 mmHg under nasal catheter oxygen inhalation, and carbon dioxide partial pressure at < 45 mmHg. The patients with hypertension and heart disease continued their antihypertensive medications on the day of surgery. For patients using the anticoagulant drug clopidogrel, clopidogrel was discontinued seven days before surgery. A low dose of aspirin (< 200 mg) is permissible for neuraxial procedures, providing a thorough risk-benefit analysis that supports its use [12]. Patients receiving low-molecular-weight heparin underwent intraspinal anesthesia 12 h after drug withdrawal and continued to use it 12 h after surgery. A filter screen was installed if deep venous thrombosis (DVT) of the lower extremity was present. Postoperative treatments included anti-osteoporosis drugs such as diphosphonate, polypeptide hormones, and monoclonal antibodies. All patients underwent perioperative thromboprophylaxis and pain management according to standardized protocols based on established guidelines, including those from the American Academy of Orthopaedic Surgeons (AAOS) [13] and the Enhanced Recovery After Surgery (ERAS) Society [14].

### Anesthesia

**Fig. 1:**
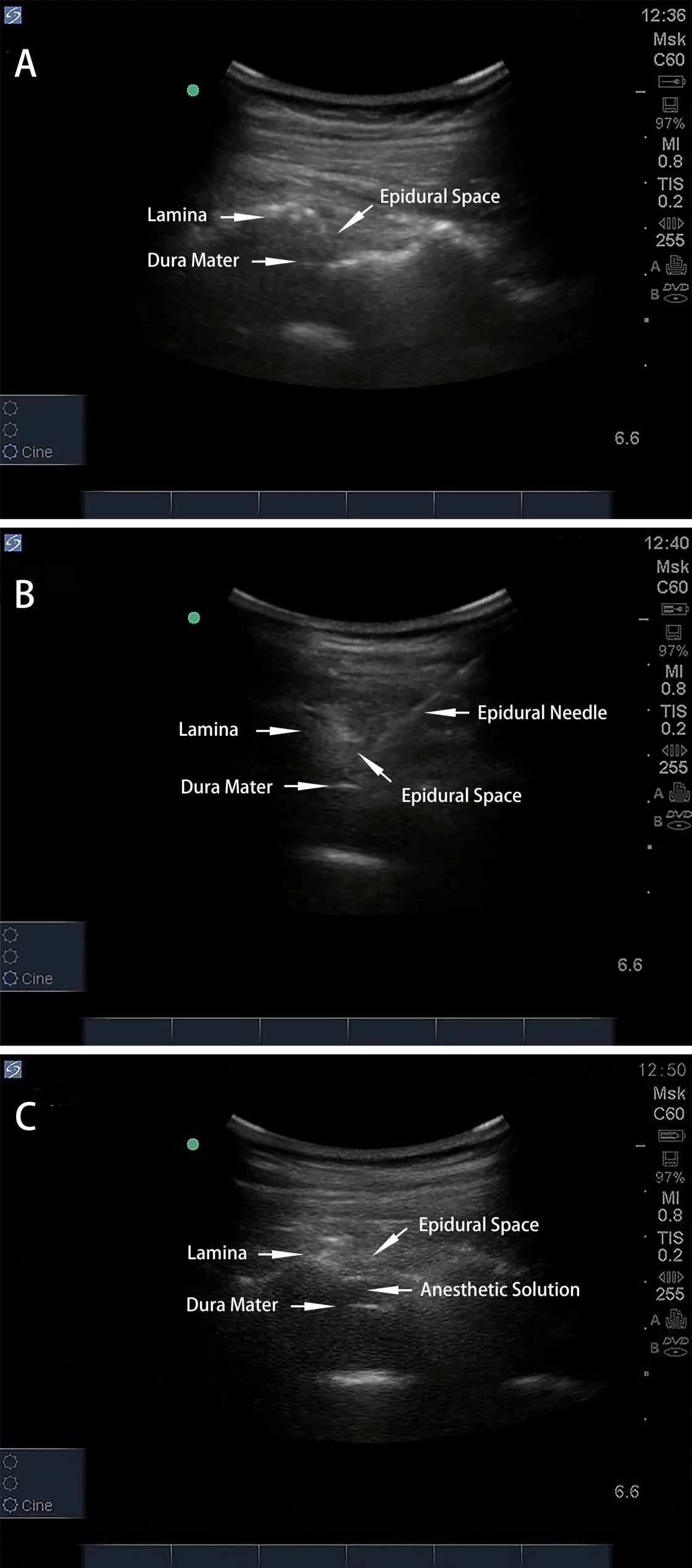
Ultrasound-guided unilateral epidural anesthesia. Representative images illustrating the standardized unilateral epidural anesthesia (UEA) technique. Ultrasound guidance was the preferred routine approach for UEA procedures in our clinical practice. **A** Ultrasound image of the epidural space showing anatomical structures and their relationships before catheter insertion and anesthetic injection. **B** The puncture needle entering the epidural space, followed by advancement of the catheter along the needle. **C** After anesthetic injection, showing diffusion of the anesthetic within the epidural space

After being transported to the operating room, the patients were routinely monitored for SpO_2_ and underwent electrocardiography and radial artery invasive pressure measurement. Central venous catheterization was used to detect the blood volume and cardiac function of patients with unstable blood pressure and the need for a large quantity of fluids over a short period, and a mask was used to provide oxygen. Ultrasound guidance was the preferred routine approach for UEA procedures to confirm needle placement, catheter position and local anesthetic spread. In the UEA group, the CSEA puncture kit (Jiangsu Yaguang Medical Instrument Co., Ltd., specification AS-E/S type II) was used for the procedure, which was conducted as follows: ① the patient was positioned with the affected side up the knee chest position with routine disinfection and draping, and 4 mL of 1% lidocaine was administered for local anesthesia at the lumbar 3–4 vertebral segment. ② The median approach was used, and the puncture was performed layer-by-layer. ③ A negative pressure test was conducted to ensure that the bubbles in the empty needle tube are compressed. ④ After needle withdrawal, no cerebrospinal fluid was aspirated, and saline injection was resistance-free (Fig. 1A). The epidural catheter was then advanced 3 cm into the epidural space along the needle (Fig. 1B). ⑤ A mixed liquid of 4 mL (8 mL of 1% ropivacaine mixed with 2 mL sterile water) was injected into the epidural space (Fig. 1C). Sensory and motor block were assessed at 5, 10, 15, and 20 min after injection. The level of anesthesia progressed from L1–2 to T10–11. During this process, asymmetrical block characteristics were observed, including differences in temperature and pain perception between the two lower limbs, followed by weaker motor function in the affected limb compared with the contralateral side. Ultimately, motor strength in the affected limb reached grade 0, while motor function on the contralateral side was relatively preserved. During surgery, 2 mL of the mixed solution was administered periodically to meet anesthesia requirements.

In the CSEA group the same processes used in the UEA group were utilized up to “④,” then: ⑤ Spinal anesthesia trocar was used to penetrate the dura mater, and it was confirmed that cerebrospinal fluid flowed out. ⑥ A 3.0 mL mixture (1.5 mL 1% ropivacaine + 1.5 mL saline) was prepared, and 2.5 mL of this mixture was injected intrathecally. The dose was adjusted with supplementary 4 mL 1% ropivacaine via the epidural route as needed. ⑦ The patient was moved into a supine position and observed for several minutes until the plane reached thoracic 10–11 and stabilized. If blood pressure dropped, 3 mg ephedrine (if heart rate was reduced) or 1 mg noradrenaline (if heart rate was normal) was administered.

### Anesthesia monitoring and evaluation

Main outcome measures: Intraoperative hemodynamic stability, including systolic blood pressure (SBP), heart rate, and SpO_2_. These parameters were analyzed via repeated-measures ANOVA to evaluate the group effect and time-by-group interaction, and between-group comparisons were performed at each observation time point. The group effect for each of the above hemodynamic parameters was set as the key endpoint. Secondary outcome measures: Time of anesthesia process; onset time of affected limb sensory block; recovery time of affected limb sensory function; rates of vasopressor and sedative use; perioperative complications (hypotension, hypoxemia, nausea and vomiting, arrhythmia, myocardial infarction, heart failure, pneumonia, respiratory failure, urinary retention, urinary tract infection, deep venous thrombosis, cognitive dysfunction, cerebrovascular events, phantom limb pain); postoperative hospital stay duration; intensive care unit admission; 30-day mortality; follow-up duration; and postoperative lower limb functional recovery assessed by the Harris hip score [15].

### Surgical procedure

Patients with femoral neck fractures underwent either total hip arthroplasty or hemiarthroplasty. Patients with intertrochanteric or subtrochanteric fractures underwent intramedullary nailing or dynamic hip screw fixation.

### Statistical analyses

Continuous variables are expressed as mean ± standard deviation. The Shapiro–Wilk test was used to assess normality, and Levene’s test was applied to evaluate homogeneity of variances. Differences in means between groups were evaluated using independent-samples t-tests. Repeated-measures ANOVA was performed to account for within-subject correlations, followed by post hoc comparisons with appropriate correction for multiple testing (Benjamini–Hochberg adjustment). Categorical variables were compared using the chi-square test, and Fisher’s exact test was applied when expected cell counts were < 5. All analyses were performed using IBM SPSS Statistics (version 16.0; SPSS UK Ltd., Woking, United Kingdom), with a significance level of *P* < 0.05. Minimum required sample size and statistical power were calculated using G*Power (University of Düsseldorf, Düsseldorf, Germany) based on an independent-samples t-test for two groups with α = 0.05 and an assumed effect size (Cohen’s d = 0.5). Post-hoc power analysis was performed to evaluate the study’s ability to detect the specified effect size.

## Results

The minimum required sample size per group was 32. A post-hoc power analysis, based on the achieved sample size and an assumed effect size (Cohen’s d = 0.5), demonstrated approximately 72% power to detect the specified difference. There were no significant differences in age, sex, number of comorbidities, body mass index, composition ratio of fracture types, surgery mode, American Society of Anesthesiologists Physical Status classification, or time of fracture onset between the two groups (all *P* > 0.05) (Table 1). The mean duration of the anesthesia procedure was 19.24 ± 4.82 min in the UEA group (95% CI: 17.74–20.74 min) and 16.47 ± 3.28 min in the CSEA group (95% CI: 15.65–17.29 min) (*P* < 0.05). The respective mean durations of affected limb sensory block and recovery were 96.05 ± 7.48 min (95% CI: 93.72–98.38 min) and 269.57 ± 27.68 min (95% CI: 260.95–278.20 min) in the UEA group and 93.23 ± 7.94 min (95% CI: 91.25–95.22 min) and 280.08 ± 34.71 min (95% CI: 271.41–288.75 min) in the CSEA group (both *P* > 0.05).

**Fig. 2:**
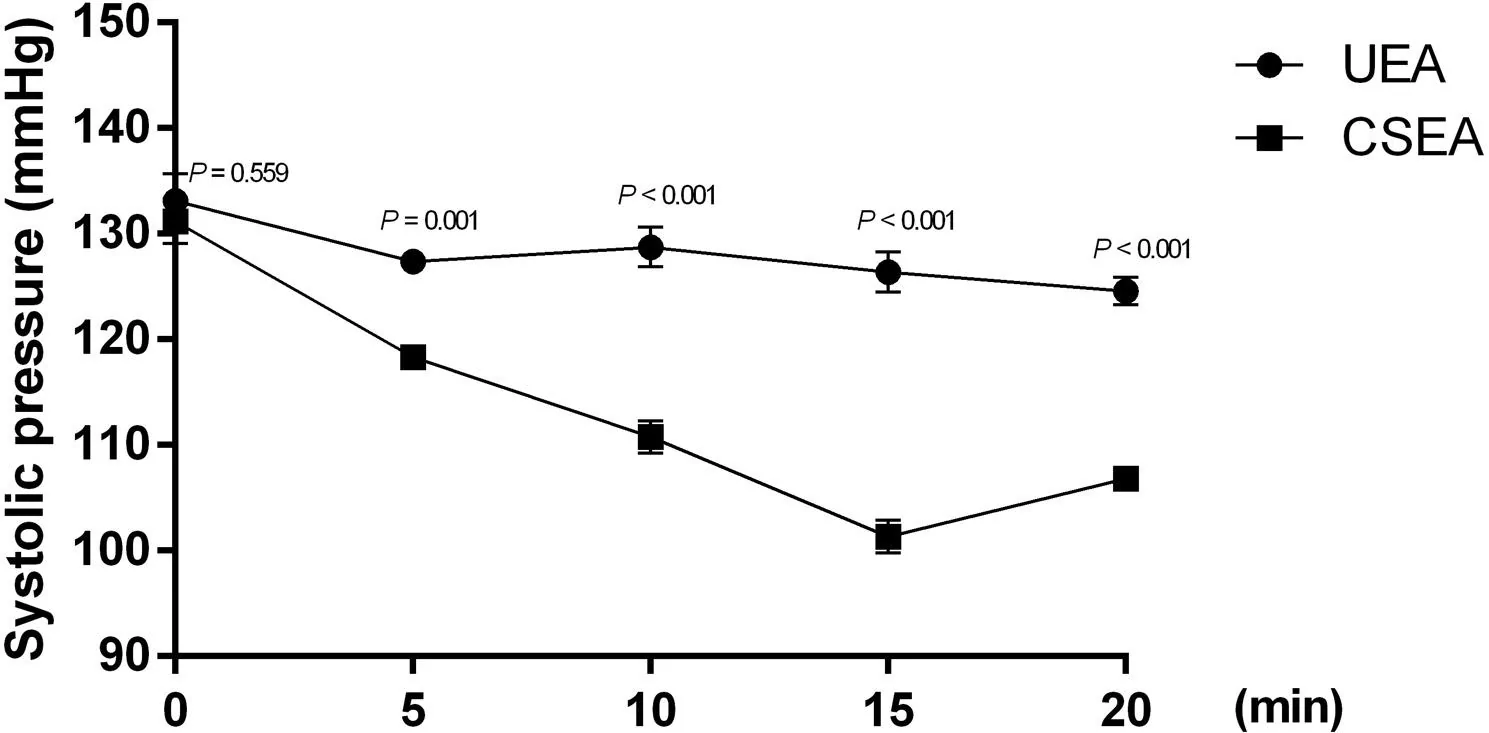
Hemodynamic trends of patients. Hemodynamic changes in systolic blood pressure over time in older patients with hip fracture receiving unilateral epidural anesthesia (UEA) or combined spinal–epidural anesthesia (CSEA). Each point represents the mean value, and the error bars indicate the standard error of the mean. All *P* values shown are unadjusted between-group values. Benjamini–Hochberg adjusted *P* values are listed in Table 2. For values extremely close to zero, *P* < 0.001 is presented uniformly

There was a significant difference in SBP between the two groups at all post-anesthesia time points (5, 10, 15, and 20 min) (all *P* < 0.05) (Table 2; Fig. 2). Vasopressor use was higher in the CSEA group (*P* < 0.05); however, this difference did not remain significant after Benjamini–Hochberg correction (adjusted *P* > 0.05). Hypotension (SBP decrease ≥ 20% from baseline or SBP < 90 mmHg) was more frequent in the CSEA group (*P* < 0.05); however, this difference did not remain significant after Benjamini–Hochberg correction (adjusted *P* > 0.05). Urinary retention and phantom limb sensation were more common in the CSEA group (both *P* < 0.05); however, these differences did not remain significant after Benjamini–Hochberg correction (both adjusted *P* > 0.05). Phantom limb sensation was identified from patient-reported symptoms documented in intraoperative and postoperative medical records. Additionally, there were no significant differences in sedative use, hospital stay, critical care admission, mortality, or lower limb function between the two groups (all *P* > 0.05) (Table 3).

**Table 2:** Hemodynamic changes preanesthesia and postanesthesia at various time-points in the two groups

Observation target	Time	UEA(*n* = 42)	CSEA(*n* = 64)	t	*P*	Adjusted-*P*	F_time_	*P*	F_group_	*P*	F_time*group_	*P*
Systolic pressure (mmHg)	Pre-anesthesia	133.10 ± 16.68	131.17 ± 16.44^bcde^	0.586	0.559	0.559	31.359	< 0.001*	185.513	< 0.001*	12.136	< 0.001*
	Post-anesthesia (5th min)	127.38 ± 6.90	118.34 ± 16.06^acde^	3.441	< 0.001*	0.001*						
	Post-anesthesia (10th min)	128.74 ± 12.15	110.75 ± 12.85^abd^	7.202	< 0.001*	< 0.001*						
	Post-anesthesia (15th min)	126.38 ± 12.36	101.31 ± 8.78^abc^	12.208	< 0.001*	< 0.001*						
	Post-anesthesia (20th min)	124.57 ± 8.50	106.80 ± 10.96^ab^	8.894	< 0.001*	< 0.001*						
Heart rate (times/min)	Pre-anesthesia	81.85 ± 10.95	78.05 ± 9.47	1.903	0.060	0.299	2.070	0.092	2.473	0.119	1.654	0.657
	Post-anesthesia (5th min)	79.10 ± 6.99	77.48 ± 6.91	1.169	0.245	0.429						
	Post-anesthesia (10th min)	76.43 ± 7.93	77.98 ± 6.09	-1.139	0.257	0.429						
	Post-anesthesia (15th min)	78.26 ± 6.60	77.09 ± 6.36	0.911	0.364	0.456						
	Post-anesthesia (20th min)	79.21 ± 6.77	78.81 ± 7.70	0.275	0.784	0.784						
SpO_2_	Pre-anesthesia	97.29 ± 1.49^d^	97.75 ± 1.35	-1.667	0.099	0.164	5.079	< 0.001*	2.751	0.100	2.330	0.055
	Post-anesthesia (5th min)	97.55 ± 1.17^d^	98.03 ± 1.31	-1.937	0.055	0.164						
	Post-anesthesia (10th min)	97.95 ± 1.27	98.41 ± 1.22^e^	-1.846	0.068	0.164						
	Post-anesthesia (15th min)	98.38 ± 1.03^ab^	98.08 ± 1.34	1.243	0.217	0.271						
	Post-anesthesia (20th min)	97.93 ± 1.33	97.70 ± 1.38^c^	0.835	0.406	0.406						

Data are presented as mean ± standard deviation. Hemodynamic parameters were analyzed using repeated-measures analysis of variance (ANOVA). Intergroup comparisons at each time point were performed using independent-samples t-tests, corrected for multiple comparisons using the Benjamini–Hochberg procedure

*UEA* unilateral epidural anesthesia, *CSEA *combined spinal epidural anesthesia, *SpO*_2_oxygen saturation

a: *P* < 0.05 vs. pre-anesthesia; b: *P* < 0.05 vs. 5 min after anesthesia; c: *P* < 0.05 vs. 10 min after anesthesia; d: *P* < 0.05 vs. 15 min after anesthesia; e: *P* < 0.05 vs. 20 min after anesthesia; *: *P* < 0.05 between groups

**Table 3:** Indexes of anesthesia process and the outcome in patients with hip fractures

Observation target	UEA group (*n* = 42)	CSEA group (*n* = 64)	Chi-square/t	*P*	Adjusted-*P*
Time of anesthesia process (min)	19.24 ± 4.82	16.47 ± 3.28	3.521	0.001*	0.024*
Time of affected limb sensory block (min)	96.05 ± 7.48	93.23 ± 7.94	1.826	0.071	0.284
Time of affected limb sensory recovery (min)	269.57 ± 27.68	280.08 ± 34.71	-1.647	0.103	0.353
Vasopressor use (%)	4/42 (9.52)	17/64 (26.56)	4.634	0.031*	0.186
Sedative use (%)	12/42 (28.57)	16/64 (25.00)	0.166	0.683	1.000
Peri-operative complications (%)					
hypotension	5/42 (11.90)	20/64 (31.25)	5.266	0.022*	0.264
hypoxemia	6/42 (14.29)	4/64 (6.25)	--	0.189	0.504
nausea and vomiting	8/42 (19.05)	13/64 (20.31)	0.026	0.873	1.000
arrhythmia	7/42 (16.67)	8/64 (12.50)	0.362	0.547	0.875
myocardial infarction	0/42 (0)	2/64 (3.13)	--	0.517	0.886
heart failure	2/42 (4.76)	6/64 (9.38)	--	0.474	1.000
pneumonia	5/42 (11.90)	5/64 (7.81)	--	0.513	0.947
respiratory failure	3/42 (7.14)	4/64 (6.25)	--	1.000	1.000
urinary retention	2/42 (4.76)	13/64 (20.31)	5.048	0.025*	0.200
urinary tract infection	4/42 (9.52)	7/64 (10.94)	--	1.000	1.000
cognitive dysfunction	9/42 (21.43)	12/64 (18.75)	0.115	0.735	1.000
cerebrovascular events	2/42 (4.76)	0/64 (0)	--	0.155	0.465
deep venous thrombosis	1/42 (2.38)	3/64 (4.69)	--	1.000	1.000
phantom limb	5/42 (11.90)	18/64(28.13)	3.927	0.048*	0.230
Postoperative hospital stays (days)	12.28 ± 6.30	11.27 ± 4.85	0.932	0.353	0.847
Follow-up (months)	12.60 ± 4.38	12.05 ± 4.06	0.660	0.511	1.000
Intensive care unit admission (%)	13/42 (30.95)	18/64 (28.13)	0.098	0.754	1.000
Mortality within 30 days (%)	3/42 (7.14)	4/64 (6.25)	--	1.000	1.000
Harris function score			0.734	0.865	1.000
excellent (90–100)	17/42 (40.48)	23/64 (35.94)			
good (80–89)	12/42 (28.57)	16/64 (25.00)			
moderate (70–79)	8/42 (19.05)	15/64 (23.44)			
poor (< 70)	5/42 (11.90)	10/64 (15.63)			

Data are presented as mean ± standard deviation or *n* (%). Continuous variables were compared using independent-samples t-test. The Harris function score was analyzed using chi-square test for overall proportions (one χ² and *P* value). Binary variables (vasopressor use, sedative use, perioperative complications, ICU admission, 30-day mortality) were analyzed using the chi-square test or Fisher’s exact test, as appropriate. Fisher’s exact test was used when > 1/5 of cells or any cell had a theoretical frequency < 5, with test statistics denoted as “--“. All outcomes in this table were predefined as secondary outcome measures. Multiple comparisons were corrected as a single family using the Benjamini–Hochberg procedure

*UEA* unilateral epidural anesthesia, *CSEA *combined spinal epidural anesthesia

*: *P* < 0.05 between groups

## Discussion

Due to the functional degradation of various organs in the body, tolerance to trauma and surgery after hip fracture are comparatively lower in older adults, and mortality and disability rates after hip fracture are extremely high. The mortality rate within one month after surgery is 10.3%, and the mortality rate within one year is 28.6% [16, 17]. In addition to the direct effects of surgery, blood loss, and perioperative complications, the influence of anesthesia on cardiopulmonary function in older patients is an important factor [9]. The toxicity and side effects of anesthesia can cause or aggravate complications in older patients, resulting in poor prognoses. Spinal anesthesia has the advantages of lower mortality rates, DVT, postoperative cognitive impairment, and fatal pulmonary embolism [18, 19]. General anesthesia has the advantages of definite effects, shorter preoperative anesthesia preparation time, and convenient airway management during surgery [8, 20]. Therefore, the general principle is that clinicians choose an anesthetic mode that is simple, safe, and effective.

The advantages of UEA include easy control of the anesthesia plane and complete regional nerve block. Numerically, less vasopressor use was observed in the UEA group. This trend failed to reach statistical significance after multiple comparison correction and warrants further prospective evaluation. This technique does not produce an absolute selective unilateral block but rather a clinically controllable, predominantly one-sided block. In the current study, blood pressure in patients who underwent UEA remained stable without large fluctuations after anesthesia; however, in the CSEA group, blood pressure decreased notably at the 15th minute after anesthesia. Moreover, compared with CSEA, UEA has a relatively narrow block region and causes less interference with physiological function. Only a small supplementary dose of epidural anesthetic is required to maintain adequate anesthesia [21], which helps minimize circulatory and respiratory fluctuations related to inadequate analgesia and may be favorable in older patients with poor compensatory capacity.

Previous studies have indicated that local anesthesia (mainly epidural anesthesia) can cause the blood vessels in the block area to dilate, and that the effective circulation blood volume is insufficient, the return blood volume is reduced, and cardiac output is reduced. In addition, older patients have a limited ability to compensate for hypovolemia induced by regional anesthesia, which makes them vulnerable to hypotension [22]. In this study, after adjustment for multiple comparisons, there were no statistically significant between-group differences in hypotension or urinary retention. Nevertheless, the UEA group had numerically fewer perioperative hypotension episodes, which may be related to limited vasodilation resulting from a narrower block range. Similarly, a numerically lower rate of urinary retention was noted in the UEA group, possibly explained by the fact that the innervation of the bladder sphincter relies on bilateral sacral nerves [23].

During regional anesthesia, an abnormal sensation caused by afferent nerve blocks is a sensation of abnormal limb positioning. This manifestation is conceptually similar to phantom limb sensation described after amputation [24]. Some patients reported feeling limb pain and a sense of restraint. Theoretically, patients who undergo CSEA are more likely to suffer from limb pain than those who undergo UEA because both lower limbs are anesthetized, which is also consistent with the findings of the current study. Numerically, fewer cases of phantom limb sensation were recorded in the UEA group, and this numerical trend warrants further prospective evaluation. In CSEA, anesthetics with heavy or equal specific gravity concentrations are used. Therefore, the patient needs to be placed in the supine position again after lumbar puncture in the lateral position, and the operation is started after anesthesia takes effect. However, UEA is very convenient in patients with hip fractures because it uses diluted local anesthetic solution, the upward affected limb in the lateral position can be directly anesthetized, and the surgery can be performed without repositioning the body. It is worth noting that the results for vasopressor use, hypotension, urinary retention, and phantom limb sensation were no longer statistically significant after correction for multiple comparisons and should therefore be interpreted with caution. However, given the relatively small sample size and limited statistical power of this study, these findings may still suggest potential trends that warrant further investigation.

Postoperative cardiac complications include myocardial infarction and heart failure. In the current study, the rate was 4.76%–9.38%, which is slightly higher than that reported in a previous study [25]. This may be because the present study included patients with a higher comorbidity ratio. Many factors affect the occurrence of perioperative cardiac complications, including old age, comorbidities, cardiac reserve, fluid volume, trauma, and surgical mode. Therefore, it is difficult to determine the direct relationship between anesthesia mode and cardiac complications. The view that the incidence of postoperative pulmonary complications in patients under general anesthesia is higher than that in patients under spinal anesthesia has been recognized by many scholars [18, 26]. Therefore, for older patients with pulmonary comorbidities, it is more reasonable to choose intraspinal anesthesia. Furthermore, for patients with poor cardiac function, UEA may be a feasible option, as this anesthesia method has a minimal impact on hemodynamic changes caused by lower limb vasodilation. Alternatively, CSEA is also a feasible solution because it is simpler in terms of the anesthetic technique.

With respect to DVT and postoperative cognitive dysfunction (POCD), Doppler ultrasonography and the Mini-Mental State Examination were used for assessment, respectively. The impact of anesthesia on the risk of DVT is complex. Anesthesia-induced vasodilation may influence venous hemodynamics in the lower extremities [27]. It is possible that differences in anesthetic distribution (e.g., predominantly unilateral versus bilateral block) may lead to asymmetrical changes in venous return. However, the development of DVT is multifactorial, and factors such as patient immobility, comorbidities, and perioperative management likely play a more important role. Moreover, the results of the current study do not reflect this difference. With the standardized use of anticoagulant drugs and venous filters, the incidence of harm due to thrombi during the perioperative period is gradually decreasing. The pathogenesis of POCD remains unclear, and it is thought that acute degenerative changes in nerve function are induced by external factors such as anesthesia or surgery on the basis of degeneration of central nerve function [28]. Previous studies have suggested that propofol, fentanyl, and ketamine (general anesthetic drugs) may induce POCD in older patients [29]. Nevertheless, the two groups of patients in the present study underwent local anesthesia, and no differences were evident in this respect. These complications often occur simultaneously or sequentially, resulting in rapid changes in the patient’s condition and even death. Therefore, adequate preoperative preparations should be undertaken in all cases to deal with possible complications so that patients can safely endure the perioperative period, although fatal complications only occur in an extreme minority of cases.

There are some differences in the treatment of femoral neck and intertrochanteric fractures, but the composition ratios of the two groups in this respect are similar. Consequently, surgical factors did not affect our conclusions. The biggest difference between the two anesthesia methods is that UEA was associated with a more stable hemodynamic profile. However, rehabilitation of hip joint function is influenced by various factors, such as surgical procedures and rehabilitation training. Therefore, anesthesia is not the only factor affecting prognosis, but it may play an important role. The fluctuation of hemodynamics in weakened older patients can cause adverse clinical events, such as shock and multiple organ failure, and it is difficult for them to fully recover body function after the stress of injury, which can lead to an increase in postoperative mortality. UEA was associated with relatively more stable hemodynamic status, consistent with its physiological rationale, and provides preliminary evidence warranting further prospective evaluation in older patients with hip fractures. Furthermore, by combining patient positioning with a diluted local anesthetic solution, our study introduces a reproducible methodological refinement that adds meaningful clinical novelty. UEA achieves predominantly unilateral spread mainly through gravity and patient lateral positioning. As acknowledged herein, differences between the two groups are multifactorial, including route of administration, anesthetic dose and concentration, and slower systemic absorption following epidural injection compared with subarachnoid administration. These factors may collectively influence the extent of sympathetic blockade and hemodynamic stability. Therefore, no direct causal inference can be drawn from the observed differences, which should be interpreted as reflecting the overall combined effects of the UEA technique rather than being attributed solely to the unilateral nature of the block. All findings should be considered exploratory and hypothesis-generating.

This study has several limitations. First, as a retrospective study, it is subject to inherent biases, including the lack of blinding of clinicians and outcome assessors, which may introduce a risk of detection bias. Second, this was a single-center study, which may limit the generalizability of the findings to other populations and clinical settings. Third, although the post-hoc statistical power was approximately 72%, which is below the conventional threshold of 80%, the study may have been underpowered to detect differences in less frequent but clinically important outcomes, such as mortality, ICU admission, and complications. Therefore, the absence of statistically significant differences in these outcomes should be interpreted with caution and should not be considered as evidence of equivalence between the two techniques. Finally, systematic quantitative assessment of contralateral sensory and motor function was not available in this retrospective study. The assessment of unilateral block was mainly based on qualitative evaluation rather than objective quantitative measurements (e.g., the exact proportion of patients with intact pinprick sensation or Bromage score 0 on the contralateral side). Consequently, the degree of unilaterality achieved cannot be precisely characterized, which limits the ability to verify the “predominantly unilateral” nature of the block. Future prospective studies should incorporate standardized neurological assessments at defined time points to address this limitation. Future large-scale, prospective, multicenter studies are warranted to further confirm and extend our findings.

## Conclusion

Compared with CSEA, UEA was associated with more stable hemodynamics. Although induction time was slightly longer, UEA may be a viable anesthetic option for older patients with hip fractures. These observed differences may reflect the combined effects of the overall technique, including variations in route of administration, anesthetic dose, and patient positioning, rather than the predominantly unilateral distribution alone. Given the retrospective, single-center, non-randomized design, all findings are exploratory and hypothesis-generating. Further prospective studies are warranted to validate these results.

## Data Availability

The datasets generated and/or analysed during the current study are available from the corresponding author on reasonable request.

## References

[1] Dent E, Daly RM, Hoogendijk EO, Scott D. Exercise to Prevent and Manage Frailty and Fragility Fractures. Curr Osteoporos Rep. 2023;21(2):205–15. 36976491 10.1007/s11914-023-00777-8 PMC10105671

[2] Yeh EJ, Rajkovic-Hooley O, Silvey M, Ambler WS, Milligan G, Pinedo-Villanueva R, et al. Impact of fragility fractures on activities of daily living and productivity in community-dwelling women: a multi-national study. Osteoporos Int. 2023;34(10):1751–62. 37335332 10.1007/s00198-023-06822-7 PMC10511617

[3] Kim BS, Lim JY, Ha YC. Recent Epidemiology of Hip Fractures in South Korea. Hip Pelvis. 2020;32(3):119–24. 32953703 10.5371/hp.2020.32.3.119 PMC7476784

[4] Chen Q, Hao P, Wong C, Zhong X, He Q, Chen Y. Development and validation of a novel nomogram of 1-year mortality in the elderly with hip fracture: a study of the MIMIC-III database. BMJ Open. 2023;13(5):e068465. 37202145 10.1136/bmjopen-2022-068465 PMC10201249

[5] Long A, Yang D, Jin L, Zhao F, Wang X, Zhang Y, et al. Admission Inflammation Markers Influence Long-term Mortality in Elderly Patients Undergoing Hip Fracture Surgery: A Retrospective Cohort Study. Orthop Surg. 2024;16(1):38–46. 37984859 10.1111/os.13932 PMC10782247

[6] Frenkel Rutenberg T, Assaly A, Vitenberg M, Shemesh S, Burg A, Haviv B, et al. Outcome of non-surgical treatment of proximal femur fractures in the fragile elderly population. Injury. 2019;50(7):1347–52. 31142435 10.1016/j.injury.2019.05.022

[7] Amrayev S, AbuJazar U, Stucinskas J, Smailys A, Tarasevicius S. Outcomes and mortality after hip fractures treated in Kazakhstan. Hip Int. 2018;28(2):205–9. 29890912 10.1177/1120700018773395

[8] Neuman MD, Feng R, Carson JL, Gaskins LJ, Dillane D, Sessler DI, et al. Spinal Anesthesia or General Anesthesia for Hip Surgery in Older Adults. N Engl J Med. 2021;385(22):2025–35. 34623788 10.1056/NEJMoa2113514

[9] Zhang G, Chen H, Zha J, Zhang J, Di J, Wang X, et al. Effect of general vs. regional anesthesia on mortality, complications, and prognosis in older adults undergoing hip fracture surgery: a propensity-score-matched cohort analysis. J Clin Med. 2022;12(1):1–9. 10.3390/jcm12010080 PMC9821019 36614881

[10] Liu Y, Su M, Li W, Yuan H, Yang C. Comparison of general anesthesia with endotracheal intubation, combined spinal-epidural anesthesia, and general anesthesia with laryngeal mask airway and nerve block for intertrochanteric fracture surgeries in elderly patients: a retrospective cohort study. BMC Anesthesiol. 2019;19(1):230. 31847846 10.1186/s12871-019-0908-2 PMC6916001

[11] Hashemi M, Dadkhah P, Taheri M, Momenzadeh S, Parsa T, Hosseini B, et al. Unilateral Epidural Blockade for Lower Limb Fracture Surgery: Parasagittal Epidural Versus Midline Epidural Anesthesia. Bull Emerg trauma. 2019;7(2):150–5. 31198804 10.29252/beat-070210 PMC6555211

[12] Kietaibl S, Ferrandis R, Godier A, Llau J, Lobo C, Macfarlane AJ, et al. Regional anaesthesia in patients on antithrombotic drugs: Joint ESAIC/ESRA guidelines. Eur J Anaesthesiol. 2022;39(2):100–32. 34980845 10.1097/EJA.0000000000001600

[13] Switzer JA, O’Connor MI. AAOS Management of Hip Fractures in Older Adults Evidence-based Clinical Practice Guideline. J Am Acad Orthop Surg. 2022;30(20):e1297–301. 36200818 10.5435/JAAOS-D-22-00273

[14] Wainwright TW, Gill M, McDonald DA, Middleton RG, Reed M, Sahota O, et al. Consensus statement for perioperative care in total hip replacement and total knee replacement surgery: Enhanced Recovery After Surgery (ERAS(^®^)) Society recommendations. Acta Orthop. 2020;91(1):3–19. 31663402 10.1080/17453674.2019.1683790 PMC7006728

[15] Harris WH. Traumatic arthritis of the hip after dislocation and acetabular fractures: treatment by mold arthroplasty. An end-result study using a new method of result evaluation. J bone joint Surg Am volume. 1969;51(4):737–55. 5783851

[16] Olcay HO, Emektar E, Ozturk ZS, Akkan S, Cevik Y. Association of Serum Lactate Levels Measured in the Emergency Department with 30-Day Mortality in Elderly Patients with Unilateral Hip Fracture. Ann Geriatr Med Res. 2024; 28 (3): 301-306. 10.4235/agmr.24.0011 PMC11467522 38952335

[17] Llopis-Cardona F, Armero C, Hurtado I, Garcia-Sempere A, Peiro S, Rodriguez-Bernal CL, et al. Incidence of Subsequent Hip Fracture and Mortality in Elderly Patients: A Multistate Population-Based Cohort Study in Eastern Spain. J Bone Min Res. 2022;37(6):1200–8. 10.1002/jbmr.4562 PMC9322522 35441744

[18] Ahn EJ, Kim HJ, Kim KW, Choi HR, Kang H, Bang SR. Comparison of general anaesthesia and regional anaesthesia in terms of mortality and complications in elderly patients with hip fracture: a nationwide population-based study. BMJ Open. 2019;9(9):e029245. 31501111 10.1136/bmjopen-2019-029245 PMC6738684

[19] Neuman Mark D, Silber Jeffrey H, Elkassabany Nabil M, Ludwig Justin M, Fleisher Lee A. Comparative Effectiveness of Regional versus General Anesthesia for Hip Fracture Surgery in Adults. Anesthesiology. 2012;117(1):72–92. 22713634 10.1097/ALN.0b013e3182545e7c

[20] Urwin SC, Parker MJ, Griffiths R. General versus regional anaesthesia for hip fracture surgery: a meta-analysis of randomized trials. Br J Anaesth. 2000;84(4):450–5. 10823094 10.1093/oxfordjournals.bja.a013468

[21] Guruvayurappan Annushha Gayathri RB, Anand Pushparani B, Gayathri, Gunaseelan Mirunalini. Effects of Differences in Epidural Needle Entry Point and Angle of Rotation of Needle Hub on the Onset and Duration of Sensory Blockade in Lower Limb Orthopaedic Surgeries: A Randomised Controlled Trial. J Clin Diagn Res. 2024;18(2):UC06–10.

[22] Hwang J, Min S, Kim C, Gil N, Kim E, Huh J. Prophylactic glycopyrrolate reduces hypotensive responses in elderly patients during spinal anesthesia: a randomized controlled trial. Can J Anaesth. 2014;61(1):32–8. 24347351 10.1007/s12630-013-0064-y

[23] Jung J, Ahn HK, Huh Y. Clinical and functional anatomy of the urethral sphincter. Int Neurourol J. 2012;16(3):102–6. 23094214 10.5213/inj.2012.16.3.102 PMC3469827

[24] Wang H, Geng Y, Zheng W, Fang W, Gu E, Liu X, et al. Phantom limb syndrome induced by combined spinal and epidural anesthesia in patients undergoing elective open gynecological surgery. Med (Baltim). 2018;97(41):e12708. 10.1097/MD.0000000000012708 PMC6203534 30313067

[25] Meyer AC, Eklund H, Hedstrom M, Modig K. The ASA score predicts infections, cardiovascular complications, and hospital readmissions after hip fracture - A nationwide cohort study. Osteoporos Int. 2021;32(11):2185–92. 34013459 10.1007/s00198-021-05956-w PMC8563539

[26] Hewson DW, Tedore TR, Hardman JG. Impact of spinal or epidural anaesthesia on perioperative outcomes in adult noncardiac surgery: a narrative review of recent evidence. Br J Anaesth. 2024;133(2):380–99. 38811298 10.1016/j.bja.2024.04.044 PMC11282476

[27] Shouhed D, Amersi F, Sibert T, Sibert K, Hemaya E, Silberman AW. Thromboprophylaxis and major oncologic surgery performed with epidural analgesia. JAMA Surg. 2013;148(1):81–4. 22987072 10.1001/2013.jamasurg.5

[28] Qin MN, Deng YN. Anesthesia-related factors in the pathogenesis of postoperative cognitive dysfunction: a mechanistic perspective. Front Neurol. 2025;16:1700911. 41561335 10.3389/fneur.2025.1700911 PMC12812579

[29] Zeng K, Long J, Li Y, Hu J. Preventing postoperative cognitive dysfunction using anesthetic drugs in elderly patients undergoing noncardiac surgery: a systematic review and meta-analysis. Int J Surg. 2023;109(1):21–31. 36799783 10.1097/JS9.0000000000000001 PMC10389238

